# The London Health Report

**Published:** 1903-01-17

**Authors:** 


### The Hospital.

A Journal of

The Medical Sciences and Hospital Administration.

Vol. XXXIII.—No. 851.] Edited by Sir Henry Burdett, K.C.B., and Solomon C. Smith, M.D., M.R.C.P. [January 17, 1903.

### THE LONDON HEALTH REPORT.

It would be difficult to imagine a more comprehensive record of unobtrusive work for the public good, not to say of work which is often unappreciated as well as unobtrusive, than is furnished every year by the Annual Report of the Medical Officer of Health of the Administrative County of London. The single fault of this report, and one which arises from the necessities of the case, is that it does not appear until the business to which it chiefly relates has been pushed out of recollection by the pressure of subsequent events. The report for 1901, for example, which has just been issued, in a volume of some hundred and fifty closely printed folio pages, contains among other matter, an account of the beginnings of the epidemic of small-pox which did not attain its full proportions until the following year, and of which the later history will be more important than the small portion which is so far unfolded to the reader. Even this portion, however, has afforded Mr. Shirley Murphy an opportunity of displaying, in a form well calculated to attract the eye, and through it to reach the minds of people who would be impervious to ordinary statistics, an illustration of the protective effects of well-performed vaccination and re-vaccination. He has given a chart of five diagrams, showing the small-pox mortality of a series of years in London, Paris, Berlin, Vienna, and St. Petersburg, and thus exhibiting at a glance a degree of immunity which reaches the highest point in Berlin, and is graded off in the other four cities in precise proportion to the efficacy with which vaccination has been performed.

It is a well-established truth that an authority of any kind is likely to be invoked in precise proportion to its diligence in the performance of its duties. When the authority is supine, those who are placed under its sway soon learn the inutility of applications to it, and refrain from any useless expenditure of their time. No sane person worries the circumlocution office. Regarded from this point of view it is eminently satisfactory to learn that the London County Council, in 1901, received no fewer than 800 applications for assistance in securing the removal of insanitary conditions. In cases in which representation had not already been made to the sanitary authority the applicants were advised to make such representation. In other cases the sanitary authorities were communicated with. *In all cases* the matter was kept under observation until the conditions complained of were remedied. In connection with these applications, 835 inspections were made by the Council's officers, and 1,463 premises were visited. It is, unfortunately, not possible to add that either the local sanitary authorities or the Local Government Board have displayed an equal readiness to grapple with the questions of administration which devolve upon them. Thus, in relation to the administration of by-laws under Section 94 of the Public Health (London) Act for houses let in lodgings, Mr. Murphy is only able to say that "on the whole" there is "some" improvement. When he proceeds to detail, it appears that difficulties have arisen as to the meaning of the word "landlord," in relation to the responsibility for the preservation of relative cleanliness and decency. The supposed "landlord" of the "lodger" is often himself only a weekly tenant, too poor to carry out the requirements of the authority; and, in these circumstances, it has been proposed to cast the responsibility upon the "owner," that is to say, upon the "landlord" of the weekly tenant instead of upon the "landlord" of the lodger. At Fulham, we are told, the late vestry framed new by-laws in order to meet this difficulty, and the Local Government Board, after some amendments, intimated their readiness to sanction them. At this stage the new municipality appeared upon the scene, and its Public Health Committee recommended the Borough Council to adopt the amended by-laws and to apply to the Local Government Board to confirm them. This the Council, which so far appears to be Vestry "writ small" rather than "large," has refused to do, with the result that the law is practically in abeyance. In other cases, as at Kensington and at Westminster, new by-laws in connection with this matter have been adopted by the respective Councils; but the Local Government Board has "not yet" given its consent, or its "final views" have "not yet" been received. In the meanwhile, the families *Cimex*, *Pulex*, and *Pediculus*, are enjoying a lively time. With reference to the still lower form of dwelling accommodation comprised under the term "common lodging houses," the County Council has been displaying laudable activity. Much attention has been devoted by the Council's officers to the maintenance of the houses in a condition free from noxious insects; and Mr. Murphy especially mentions the use for this purpose of a plumber's blowpipe, the flame from which is able to penetrate the interstices of iron bedsteads and of woodwork. Additional evidence of improvement is afforded by the diminished number of prosecutions instituted by the Council. We have no space in which to follow the rest of this highly interesting and important record; but we cannot leave it without offering our hearty congratulations to Mr. Shirley Murphy upon the value and the excellence of his work.

